# The impact of cannabis use on ageing and longevity: a systematic review of research insights

**DOI:** 10.1186/s42238-025-00267-x

**Published:** 2025-07-29

**Authors:** Sonam Nain, Niraj Singh, Anne Katrin Schlag, Michael Barnes

**Affiliations:** 1 Source Bioscience, Endeavour House, Cambridge, CB24 9ZR UK; 2 Lenus Global, 1-7 Harley Street, London, W1G 9QD UK; 3 Drug Science, Camburgh House, 27 New Dover Rd, Canterbury, CT1 3DN UK; 4 Maple Tree Consultants, Newcastle, NE3 4JQ UK

**Keywords:** Cannabis, Cannabidiol, CBD, Delta-9-tetrahydrocannabinol, THC, Anti-ageing, Longevity, Experimental models, Lifespan, Clinical population

## Abstract

**Background:**

With aging emerging as a global challenge linked to chronic diseases, identifying interventions that support a healthy lifespan and health span has become imperative. Cannabinoids derived from cannabis, particularly cannabidiol (CBD) and Δ9-tetrahydrocannabinol (THC), have gained attention for their potential to promote healthy aging through interactions with the endocannabinoid system. While CBD has often been highlighted for its benefits, emerging evidence indicates that THC, under certain conditions and doses, may also play a therapeutic role in aging. Despite this interest, significant knowledge gaps persist in understanding cannabis’s role in promoting healthy aging and longevity.

**Aim:**

We reviewed recent literature to investigate the effect of cannabinoid use, particularly CBD and THC on aging and longevity. By synthesizing findings from preclinical models, clinical studies, and real-world evidence, we aimed to elucidate the potential of cannabinoids, in fostering healthy aging, mitigate age-related decline, and promote well-being in older populations.

**Method:**

We conducted a systematic review guided by PRISMA to investigate the impact of cannabinoids on aging and longevity. Studies involving preclinical models (e.g., *Caenorhabditis elegans*, rodents, zebrafish, and mice) and clinical populations aged 50 years and older were included. Exclusion criteria targeted acute effects and mechanisms of action in different medical conditions. Aging was explicitly defined as biological and psychological changes associated with advancing age, and longevity was defined as the extension of lifespan and factors influencing healthy aging.

**Findings:**

Eighteen studies investigating the direct impact of cannabinoids on aging and longevity were identified in preclinical models (11) and human studies (7). Preclinical studies have shown promising results regarding the potential benefits of cannabinoids including improved lifespan, cognitive function, inflammation, memory, sleep quality, and social interaction. The effects of THC appear more complex, with potential benefits at low doses and drawbacks at higher doses, highlighting their complex role in aging. However, the limited number of human studies hinders a comprehensive understanding. Clinical studies also suggest potential therapeutic applications for cannabinoids in aging populations, although further research is needed to understand their mechanisms of action and long-term effects fully.

**Conclusion:**

Cannabinoids hold promise for supporting healthy aging and enhancing the quality of life in older populations. While preliminary research suggests intriguing possibilities, more studies are needed to solidify the link between cannabis use, the ECS, and healthy aging in humans. Rigorous clinical trials are crucial to evaluate their safety and efficacy. Longitudinal studies and well-designed clinical trials are critical to understanding the safety, efficacy, and long-term effects of cannabis use in aging populations. Future research should optimize dosages, investigate mechanisms of action, and explore the influence of cannabis use initiated in aging as opposed to lifetime exposure. Clarifying these aspects is vital for informing public health strategies and developing targeted therapeutic interventions for age-related conditions.

## Introduction

The rapid aging of the global population raises significant social and economic concerns, propelling aging-related research to the forefront. Due to various advancements, life expectancy has increased dramatically, with projections estimating over 2 billion individuals will be over 60 years old by 2050, as reported by World Population Ageing in January 2023 (World Population Ageing., 2023). This demographic shift is underscored by United Nations data from February 2023 indicating a global average lifespan of 72.8 years in 2019 is expected to rise to 77.2 years by 2050. Currently, 10% of the world’s population is over 65, signalling a major shift towards an aging society (United Nations, World Population Prospects, 2023). This trend presents both challenges and opportunities, particularly when addressing age-related health issues such as Alzheimer’s disease, cardiovascular disorders, cancer, and more. The incidence of these diseases effectively doubles every 5 years after the age of 60 (Melzer et al. 2020). In response to these challenges, scientific inquiries have intensified, focusing on unravelling the intricate cellular and molecular mechanisms underpinning aging and longevity.

Aging itself is a multifaceted phenomenon encompassing a wide array of physiological transformations that significantly impact an individual’s health and well-being. As we age, we often experience declines in cognitive function, altered metabolic processes, and an increased susceptibility to various diseases and disorders (DiLoreto and Murphy 2015; Partridge 2018). These age-related changes arise from a complex interplay of genetic factors, environmental influences, and lifestyle choices, ultimately contributing to an increased vulnerability to illness and mortality. In recent years, researchers have identified twelve distinct hallmarks of aging that characterize the biological processes underlying the aging phenomenon. These hallmarks include genomic instability, telomere attrition, epigenetic alterations, cellular senescence, dysregulated autophagy and among others (López-Otín et al. 2023; Tenchov et al. 2023). Each hallmark is interconnected, forming a complex network of mechanisms that contribute to the aging process. Additionally, proposed hallmarks of health highlight the immune system’s role, homeostasis maintenance, and stress response in promoting well-being (Tartiere et al., 2024).

Scientists are delving into various pharmacological interventions to address the hallmarks of aging and potentially mitigate or reverse its effects. Among the promising candidates are mTOR inhibitors, such as rapamycin, which function by impeding the mTOR protein responsible for regulating cell growth and proliferation. Although studies indicate its potential in slowing aging, further research is necessary to ascertain its efficacy (Blagosklonny 2023; Lamming et al. 2013; Lee et al. 2024; Selvarani et al. 2021). Sirtuin activators represent another avenue, stimulating sirtuins, pivotal proteins involved in cellular repair and stress management (Bayele 2020; Grabowska et al. 2017). Resveratrol, commonly found in red wine, exemplifies this category, although conclusive evidence of its direct anti-aging effects in humans remains elusive(Kaeberlein et al. 2005). Formin proteins, known for their role in cell structure and movement, are also under scrutiny for their potential involvement in regulating lifespan, yet their specific contribution warrants deeper investigation(DeWard et al. 2010).

Beyond these pharmacological strategies, the endocannabinoid system (ECS) has emerged as a potential player in influencing both aging and longevity (Paradisi et al. 2012). The intricate network of neurotransmitters, ECS receptors, and enzymes plays a critical role in regulating various physiological processes that are essential for healthy aging, including cell death (apoptosis), mitochondrial function, brain function, and maintaining a stable internal environment homeostasis (Sallaberry and Astern 2018). Dysregulation of the ECS has been linked to various age-related conditions, suggesting a potential link between its proper functioning and healthy aging.

Cannabis-derived compounds, such as cannabidiol (CBD) and Δ9-tetrahydrocannabinol (THC), interact with the endocannabinoid system (ECS), modulating a variety of physiological processes. These compounds have gained considerable attention for their diverse properties, which are mediated through the ECS. (Bilkei-Gorzo 2012; Haney 2022). CBD is well-known for its neuroprotective, anti-inflammatory, and antioxidant effects, making it a promising candidate for supporting brain health (Ellingson et al. 2021; Hampson et al. 1998a, b; Iseger and Bossong 2015; Kovalchuk and Kovalchuk 2020; Millar et al. 2019; Osborne et al. 2017; Simona Pisanti a 1, 2017; Winiger et al. 2021). In contrast, THC, particularly at lower doses, has shown potential for enhancing cognitive function, reducing neuroinflammation, and supporting overall brain health, especially in aged models (Chen et al. 2013; Fihurka et al. 2022a).

Beyond THC and CBD, other cannabinoids, such as cannabigerol (CBG) and cannabinol (CBN), also exhibit neuroprotective and anti-inflammatory properties, further contributing to brain health (Calapai et al., 2022; Khouchlaa et al. 2024; Li et al., 2024).These cannabinoids work synergistically with terpenes, like myrcene and linalool, which possess additional therapeutic effects such as anti-inflammatory, analgesic, and sedative properties (Del Prado-Audelo et al. 2021; Surendran et al., 2021).This synergistic interaction between cannabinoids and terpenes enhances the therapeutic potential of cannabis through the “entourage effect.” (Russo 2011).The entourage effect suggests that the combined action of these compounds is more effective than any single compound acting alone, with each compound potentially enhancing or modifying the effects of the others, leading to a more balanced and potent therapeutic profile.

Since maintaining homeostasis is crucial for a long and healthy life, research is ongoing to understand how these cannabinoids interact with ECS receptors in the brain, potentially impacting homeostasis across different systems. Studies exploring THC’s effects on endocannabinoid homeostasis, such as in placenta and cancer cells, and its involvement in cell death (apoptosis), highlight its potential for maintaining cellular balance (Maia et al. 2019; Velasco et al. 2016). Similarly, CBD’s ability to modulate the ECS, particularly its interaction with enzymes and receptors like FAAH and PPARs involved in fat and sugar metabolism, suggests promise for promoting healthy aging (Leweke et al., 2012). This research paves the way for understanding how cannabinoid modulation of homeostasis may influence longevity and anti-aging efforts in the future.

As societal perceptions of aging and cannabinoids evolve, further exploration of cannabinoids such as CBD and THC applications, their effects on aging-related markers and signalling pathways holds immense potential for promoting healthy aging and potentially extending the human health span. This comprehensive review article embarks on a deep dive into the current research landscape, focusing on the impact of cannabinoids on aging and longevity within the realm of both experimental studies and human studies. Furthermore, this review will shed light on the limitations and challenges associated with employing experimental models to study aging and the requirement for more studies for translating findings to humans. By acknowledging these limitations and highlighting areas for further exploration, this review aspires to contribute significantly to the burgeoning field of anti-aging research within the context of controlled experimental environments.

## Methodology

There is no pre-registered protocol. However, we followed the Preferred Reporting Items for Systematic Reviews and Meta-analyses (Moher et al. 2009).

A comprehensive exploration of how cannabis affects aging and lifespan was conducted through a thorough PubMed and Scopus search PubMed and Scopus databases from the last 15 years covering the period from January 2008 to February 2023. Keywords such as “Cannabis” OR “Cannabidiol” OR “Cannabinoid” OR “CBD” OR “Marijuana” OR “Marihuana” OR “tetrahydrocannabinol” OR “delta-9-tetrahydrocannabinol” OR “THC” AND “Aging” OR “Longevity” OR “old adults” OR “old age” OR “lifespan,” OR “health span” OR “preclinical studies” OR “Animal studies” OR “human studies” were utilized.

The search and data abstraction was conducted by SN, and studies with potential relevance underwent further review by the other authors for inclusion. Studies were included if they specifically examined the direct use or dosage of cannabinoids in aging and longevity. We targeted studies that specifically examined the use of cannabis during aging or long-term cannabis use over the years, with participants using cannabis at least once a week while aging. Studies focusing on the acute effects of cannabis or its mechanisms of action in medical conditions such as chronic pain, multiple sclerosis, epilepsy, nausea, HIV, and psychiatric disorders were excluded.

Additionally, the age of studies was restricted, with a focus on the older population in human studies. However, human studies on cannabis use and aging share common methodological issues, including inconsistent reporting of dosage, with cannabis use often described in broad terms like “heavy” or “recreational” use, without precise measurements. Age categories are often generalized (e.g., “older adults”) without clear subgroups, making it difficult to draw specific conclusions. These inconsistencies hinder the clarity and comparability of the findings. Nevertheless, we attempted to combine these studies to get a better understanding of the overall trends and effects of cannabis use in aging populations.

Additionally, real-world evidence on Cannabis-Based Medicinal Products (CBMPs) was explored to understand their potential in addressing aging-related challenges. However, the literature review was limited to reports published in the English language.

## Results

A total of 2,348 articles were initially identified through database searches (PubMed and Scopus), covering a wide range of topics related to cannabis use. These topics included its mechanisms of action, medical applications, aging, recreational use patterns, societal impacts, neurocognitive effects, legal and regulatory considerations, public health implications, and potential therapeutic roles in conditions like chronic pain, multiple sclerosis, epilepsy, nausea, and psychiatric disorders. These articles were subsequently screened for relevance to the intersection of cannabis, aging, and underlying mechanisms. From this process, 309 articles were selected for further evaluation. An additional six articles were identified through forward searching, which involved reviewing more recent publications that cited the initially relevant studies. One study was inaccessible and thus excluded. This approach ensured the inclusion of newer or related research that may have been missed in the original search.

**Fig. 1:**
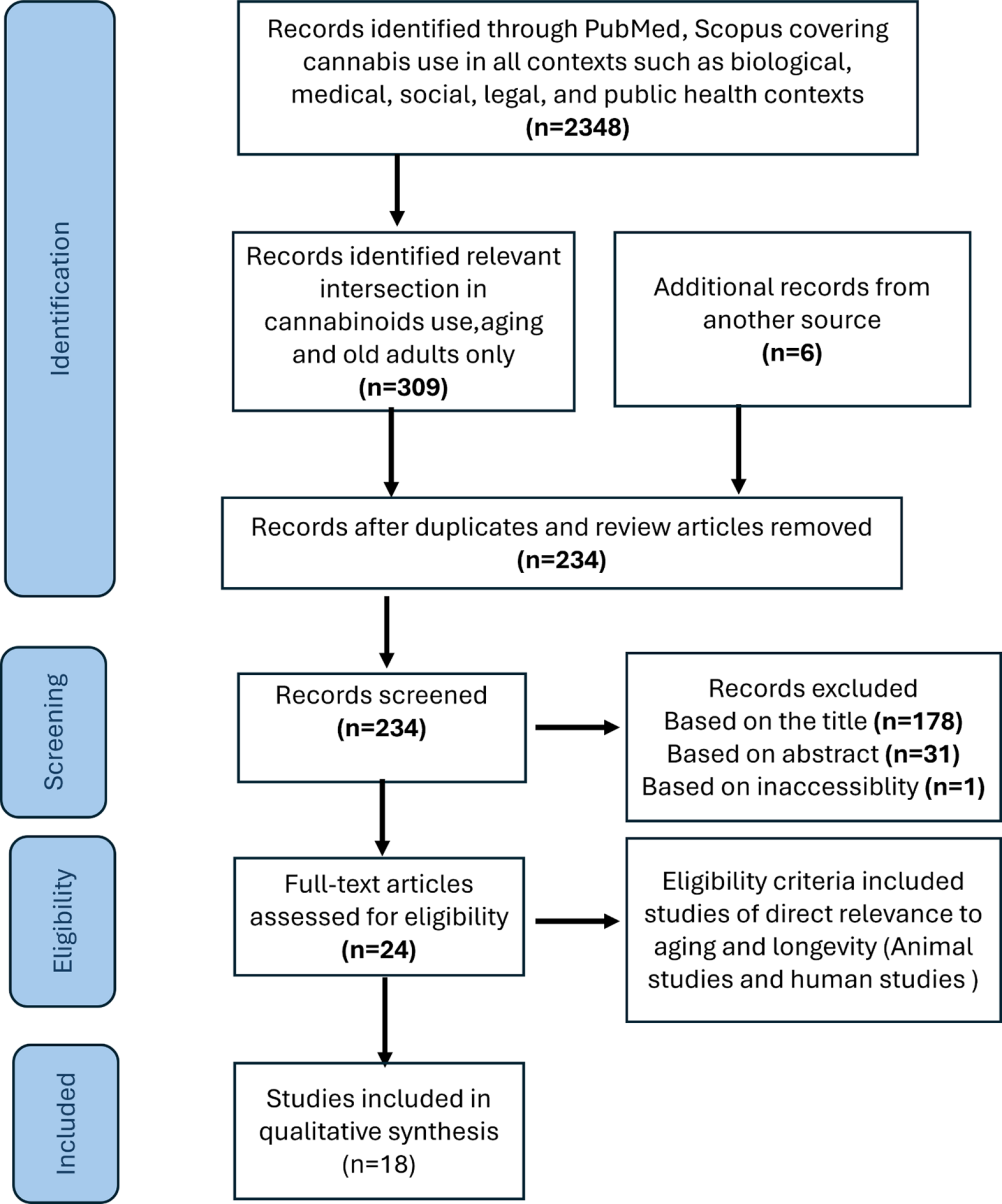
PRISMA flowchart of literature search. n = number of records, PRISMA = Preferred Reporting Items for Systematic Reviews and Meta-Analyses (Moher et al. 2009)

While most studies focused primarily on cannabis’s mechanisms of action in various medical conditions,24 studies were specifically included due to their focus on the use of cannabinoids in aging. Among these 24 studies, 18 explored the impact of cannabis on aging and lifespan. These studies comprised 11 preclinical models and 7 human studies (Fig. 1) and (Tables 1 and 2).

**Table 1:** Preclinical studies examining the relationship between cannabis use and its impact on aging

Experimental models	CBD dosage	THC dosage	Mode of administration	Impact on antiaging	References
***C. elegans***	10–100 μm	NA	oral administration via liquid NGM mixed with CBD and DMSO.	Increased lifespan by 12.2–18.3%, increased resistance to heat-induced molecular stress, high conc. of CBD (4000 μm) had a significant negative effect on motility	(Land et al. 2021)
	1–10 µM	NA	oral administration	Induced neuronal autophagy, enhancing lifespan through induction of autophagy genes and SIRT1.	Wang et al. 2022)
**Zebrafish**	0.02–0.5 µM	NA	Waterborne exposure	Exposure at embryonic level increased the survival and reduces size	(Pandelides et al. 2020a)
	NA	0.08- 2 µM	Waterborne exposure	Embryonic-larval exposure to 0.08 µM THC enhanced survival, but elevating THC levels to 2 µM diminished this protective effect.	(Pandelides et al. 2020b)
***Drosophila melanogaster***	0.1 or 3 µM	0.1 or 3 µM	Oral ingestion	Significantly increased longevity at 3 µM following trauma but THC did not quite reach any significance. Both CBD and THC displayed neuro-protective properties	(Candib et al. 2023)
**Mice**	NA	3 mg per kg per bodyweight	Oral ingestion	Low dose of THC reversed the cognitive decline in the older mice and promotes longevity	(Bilkei-Gorzo et al. 2017)
	NA	0.002 and 0.002 mg/kg mg/kg	Osmotic pump	Ultra-low THC also improved memory and learning functions similar to the high dose of THC	(Komorowska-Müller et al. 2023)
	NA	0.002 mg/kg bodyweight	Intraperitoneal injection	Increased cognitive performance in female mice	(Sarne et al. 2018)
	NA	0.002 and 0.02 mg per kg per bodyweight	Intranasal injection	THC intranasal treatment slows down the memory decline and promotes healthy lifespan	(Fihurka et al. 2022a)
	10 mg per kg per day	NA	Oral ingestion	CBD combine with omega-3 fatty acids in high-fat diet mice revealed prolonged lifespan and potential neuroprotective effects	(Abi et al. 2022)
	NA	3 mg per kg per bodyweight	Intraperitoneal injection.	THC alone increased cognitive performance, while the combination of CBD and THC (1 mg/kg/day each) did not yield the same effect.	(Nidadavolu et al. 2021)

CBD: Cannabidiol (CBD) THC: Δ9-tetrahydrocannabinol

**Table 2:** Human studies on cannabis use in older adults and their outcomes related to anti-aging effects

Age group	Study design	Impact on antiaging	References
60 + years	Aged 60 years and older who reported consuming cannabis at least once per week for at least the last year (*n* = 28) or who reported never using cannabis (*n* = 28). Lifetime users had an average of 23.55 years of cannabis use.	**Negative**: Lifetime users displayed inferior executive function and general cognition compared to others.	(Thayer et al. 2019)
43–55 + years	A cohort of black and white men and women, aged 18–30, followed for 25 years to assess cumulative marijuana exposure and cognitive function.	**Mixed**: Past use linked to poorer verbal memory, but no impact on other cognitive functions assessed.	(Auer et al. 2016)
66.6 years (avg.)	Early-life cannabis vs. nonusers. Investigated morphological differences in hippocampal subregions in older adults with a history of early life canabis use.	**Negative**: Exacerbated age-related cognitive decline in early-life users.	(Burggren et al. 2018)
60 years of age or older	Resting state functional connectivity (rsFC) compared between cannabis users (*n* = 43) and non-users (*n* = 153).	**Possible positive**: Increased functional connectivity observed, suggesting potential mitigation of age-related cognitive decline.	(Watson et al., 2022)
60 + years (Current users)	Longitudinal study of older adults who reported cannabis use at different ages.	**Neutral**: Cannabis use is reported as an alternative therapy, with reasons including age-related changes, policy shifts, and non-smoking options.	(Arora et al. 2021)
Followed till 45 years	Long-term cannabis users (*n* = 86), used cannabis weekly or more frequently at age 45 years, or were dependent on cannabis.	**Negative**: Accelerated biological aging and poorer midlife health, financial, and social preparedness for late life.	(Meier et al. 2022)
60 + years (Small sample size)	Small sample size (28 cannabis users and 10 non-users).	**Negative**: Long-term cannabis users performed lower in cognitive domains compared to non-users and short-term users.	(Stypulkowski and Thayer 2022)

## Cannabinoids and anti-aging

The increasing use of cannabinoids, particularly CBD and THC, by older adults presents a complex scenario. While anecdotal evidence and preliminary research suggest potential benefits, a cautious approach is essential due to limited large-scale human studies. A University of Iowa study examining 14,896 respondents reported that cannabis use in adults aged 50–64 increased from 9 to 17%, while use among those aged 65 + years rose from 2.4 to 4.2% over the study period.(Han and Palamar 2020).In Canada, where legalization has influenced usage, 15.5% of 9,766 participants (average age 73.2 years) reported cannabis use, primarily for chronic conditions such as arthritis and pain. Among these, 7.1% cited cannabis use in the past year, reflecting a significant rise. Respondents frequently noted chronic pain, sleep disorders, and mental health issues like anxiety and depression as key reasons for use. This trend underscores the need for further investigation into its therapeutic potential and risks. (Tumati et al. 2022).

Currently, Canadian guidelines support the use of cannabis for a limited number of conditions in all age ranges when standard treatments are ineffective (e.g., neuropathic, and palliative pain, chemotherapy-induced nausea and vomiting, spasticity in multiple sclerosis and spinal cord injury) (Allan et al. 2018). Surveys indicate older adults use cannabis for pain management, improved sleep, and managing anxiety and depression (Alex and Kleidon 2023) research on cannabinoids and aging is in its early stages. Preclinical studies using cells and animals offer promising signs that cannabinoids may possess anti-inflammatory and antioxidant properties, potentially beneficial for age-related issues. These preclinical findings haven’t translated into large-scale human trials specifically designed to investigate the anti-aging effects of cannabinoids. Most existing human studies are observational and unable to establish causality between cannabis use and health outcomes in older adults.

### Preclinical studies

Several pre-clinical studies have utilized diverse invertebrate and rodent models to explore the impact of cannabinoids on aging and longevity, showcasing a progression of research findings over the years. Table 1 provides a comprehensive summary of the diverse experimental designs utilized in preclinical studies across various models, elucidating the observed impacts on aging and longevity.

#### Caenorhabditis elegans (C. Elegans)

*C. elegans* serves as a pivotal model organism for preclinical lifelong toxicity studies due to its short lifespan of 2–3 weeks and the significant overlap of its genes with those of humans, estimated to be between 60 and 80% (Wood William 1998; Zhang et al. 2020). In a study by Land et al. (2021), long-term exposure to CBD at concentrations ranging from 10 to 100 µM resulted in an 18% increase in maximum lifespan and enhanced motility during late-stage life. Importantly, this study found no evidence of acute toxicity across a wide range of concentrations (0.4 µM to 4 mM), except for a decrease in motility observed at the highest concentration of 4000 µM. CBD treatment also enhanced thermotolerance, further underlining its potential benefits for physiological functions in *C. elegans* (Land et al. 2021). In another investigation by Wang et al. (2021), CBD’s impact on neurodegeneration was explored using transgenic C. elegans expressing human amyloid-β peptide. The results showed that CBD not only extended lifespan but also improved healthspan by preventing Aβ-induced neuronal damage, a process mediated by CB1 receptor activation.(Wang et al. 2021).

Further research by (Wang et al. 2022) demonstrated that cannabinoids play a critical role in promoting autophagy and improving neuronal health. They observed that cannabinoids stimulated autophagic flux in nerve-ring neurons, hippocampal cells, and SH-SY5Y neurons, contributing to anti-aging effects. This was validated through RNAi knockdown experiments targeting autophagy-related genes (bec-1, vps-34, and sqst-1). Additionally, CBD helped mitigate age-related morphological changes in touch receptor neurons (TRNs), slowing the onset of abnormal morphologies. RNAi knockdown experiments identified the involvement of specific genes (bec-1 and sqst-1) in CBD-mediated changes. Importantly, CBD-induced lifespan extension and improved neuronal health were contingent on the presence of sir-2.1/SIRT1, a key factor in longevity.(Wang et al. 2022).

#### Zebrafish

Zebrafish (*Danio rerio*) have become a prominent model for unravelling the intricate relationship between cannabinoids and aging. With shared cellular and molecular mechanisms like mammals and a lifespan of approximately 3 years, zebrafish provide a useful model for investigating aging processes in a manner comparable to humans (Adams and Kafaligonul, 2018; Beis and Agalou 2020). Their well-conserved endocannabinoid system further enhances their use in cannabinoid research (Oltrabella et al. 2017). Previous studies on cannabidiol (CBD) in zebrafish have raised concerns that significant exposure during development may result in developmental teratogenicity and alterations in gene expression. However, when exposed to lower, human-therapeutic doses, long-term changes in fecundity were observed (Carty et al. 2019).

The first study dives into the “The Developmental Origins of Health and Disease (DOHaD)” concept, examining how early exposure to CBD during embryo development affects aging across generations. Zebrafish embryos were exposed to CBD concentrations ranging from 0.02 to 0.5 µM during development. The study followed these zebrafish for 12 months, with a more detailed analysis at 30 months, assessing survival, behavior, reproduction, spinal curvature, and aging markers. While the study predicted significant changes in these parameters due to CBD exposure, the specific outcomes are still under investigation (Pandelides et al. 2020a).

The second study shifts focus to THC, exploring its long-term effects on zebrafish longevity. Similar to the CBD study, zebrafish were exposed to various THC concentrations during development, ranging from 0.08 to 2 µM. After 30 months, the researchers not only looked at the adult fish but also their offspring (F1 generation). Interestingly, the lowest THC dose (0.08 µM) showed promising results, increasing male survival and egg production in adults while reducing markers of aging and inflammation in the liver (Pandelides et al. 2020b). However, higher doses had negative consequences, impacting offspring survival and reproduction. However, these potential benefits need further investigation in other models and with different cannabinoids, considering the biphasic nature observed.

These findings underscore the complexity of cannabinoid-aging interactions in zebrafish. While detrimental effects exist, particularly with high-dose exposure, low-dose THC suggests intriguing possibilities for lifespan, inflammation, and senescence modulation.

#### Drosophila melanogaster

The human fruit fly, *Drosophila melanogaster*, proves to be a powerful model for understanding the effects of cannabis on lifespan and brain function. Their short lifespans and shared genes with humans (70%) allow researchers to quickly observe aging effects and potentially apply findings to humans. Plus, their well-mapped nervous system aids in deciphering complex brain functions (Scheffer et al. 2020). Study using Drosophila melanogaster have shown promising results for cannabis in extending lifespan and delaying age-related neurodegeneration.

A recent study by Candib et al. 2023 built upon this work by investigating how CBD or tetrahydrocannabinol (THC) impact various aspects of health in flies. Notably, a specific dose of CBD (3 µM) again significantly extended lifespan, while both CBD and THC displayed neuroprotective properties after traumatic brain injury (TBI). However, finding the optimal dosage and understanding long-term effects in different situations requires further research. They also explored the impact of CBD and THC on sleep, circadian rhythms, and age-related decline in movement. While no significant improvements were observed in sleep and circadian rhythms, it’s important to note that other studies suggest potential effects of cannabinoids in these areas warranting further exploration (Murillo-Rodríguez et al. 2006). Interestingly, the study highlighted CBD’s potential to counteract the negative effects of TBI on brain function and improve movement, suggesting its neuroprotective properties. Additionally, low-dose THC showed promising results for survival and longevity after TBI, but further research is needed to confirm these findings (Candib et al. 2023).

#### Mice

Mice share a similar endocannabinoid system (ECS) with humans, comprising cannabinoid receptors (CB1 and CB2), endocannabinoids (natural cannabinoids produced by the body), and enzymes responsible for their synthesis and degradation. This shared system allows researchers to investigate the interaction of specific cannabinoids, like THC or CBD, with the ECS, elucidating their mechanisms of action and potential therapeutic applications (Zou and Kumar 2018).

Studies in mice have revealed intriguing findings regarding the effects of THC, particularly at low doses, which vary depending on the animal’s age. In older animals, oral THC administration has been associated with increased synaptic connectivity, improved memory, and even reversal of age-related cognitive decline. Specifically, researchers observed heightened spine density in the hippocampus, a brain region critical for memory, and enhanced cognitive performance in older mice treated with THC. Conversely, younger animals treated with THC exhibited worsened cognitive performance. This underscores the significance of considering age when exploring cannabinoid applications (Bilkei-Gorzo et al. 2017).

A recent study by Komorowska et al. has also shown that chronic exposure to low doses of THC enhances memory and learning functions in mature to older mice. The study revealed that chronic THC exposure, defined as 28 days of use at doses of 3 mg/kg or lower, administered via an osmotic pump, increased the number of synapses in the hippocampus. This led to improved memory and learning functions in mature to older mice aged 12 and 18 months. (Komorowska-Müller et al. 2023). Similar findings were reported by Sarne et al., who investigated the cognitive effects of a single intraperitoneal injection of an extremely low dose of THC (0.002 mg/kg) in female mice. They observed improved performance in THC-treated older mice compared to vehicle-treated older mice, particularly in terms of learning and spatial memory (Sarne et al. 2018).

Research on CB1 receptor agonists, which target the same receptor stimulated by THC, has revealed improvements in spatial memory, reductions in age-related inflammation, and induction of hippocampal neurogenesis in old rats (Marchalant et al. 2008). These opposing effects of cannabis constituents on different age groups may be mediated by age-related changes in the endocannabinoid system, including decreased CB1 receptor binding and gene expression in key brain regions such as the cerebral cortex, limbic structures, and hippocampus (Berrendero et al. 1998). Furthermore, investigations into the chronic low-dose THC treatment in old mice have shown promising results in reversing age-related cognitive decline and restoring hippocampal gene transcription patterns, emphasizing the importance of restoring CB1 signaling to treat age-related cognitive impairments.

A Fihurka et al., study explored the potential of intranasal delivery of low-dose THC in aged mice with Alzheimer’s disease. This study found that the THC treatment in aged APP/PS1 mice preserved memory and reduced levels of Alzheimer’s disease-related proteins, suggesting its potential as a therapeutic strategy. The intranasal delivery method minimized systemic exposure, potentially reducing side effects compared to other administration methods (Fihurka et al. 2022a).

While both THC and CBD hold promise for addressing age-related cognitive decline, understanding their interactions is crucial. A recent study revealed that while low-dose THC (1 mg/kg/day) alone significantly improved spatial learning in aged mice, combining it with CBD in a 1:1 ratio did not. This suggests CBD may alter THC metabolism, causing a temporary rise in blood THC levels followed by rapid clearance, potentially diminishing the observed benefits. This highlights the need for careful consideration of cannabinoid interactions when designing therapeutic strategies (Nidadavolu et al. 2021).

Another study investigated the neuroprotective effects of CBD and omega-3 fatty acids in mice fed a chronic high-fat diet. The treatments were administered orally, with the mice receiving CBD (10 mg/kg/day) and omega-3 (200 mg/kg/day) alongside the high-fat diet for 16 weeks. The study revealed prolonged lifespan, amelioration of brain ischemia, and significant astrocytosis, indicating potential neuroprotective effects. These findings underscore the complex interplay between cannabinoids, aging, and neuroprotection, providing valuable insights into potential therapeutic interventions for age-related cognitive decline and neurological disorders. Findings also open new avenues for exploring the combined benefits of CBD and Omega-3 for promoting longevity and brain health, especially in contexts like high-fat diets (Abi et al. 2022). For a summary of preclinical studies examining the relationship between cannabis use and its impact on aging, please see Table 1.

### Human studies

To date, empirical studies regarding the effects of cannabis use in older adults are sparse. Tetrahydrocannabinol (THC) and cannabidiol (CBD) both show promise in addressing age-related health issues. THC enhances memory, reduces inflammation, and offers neuroprotection, while CBD extends lifespan, improves motility, and promotes autophagy in preclinical models(Arora et al. 2021; Bilkei-Gorzo et al. 2017; Komorowska-Müller et al. 2023; Marchalant et al. 2008; Nidadavolu et al. 2021). Both cannabinoids highlight potential for longevity and cognitive resilience, though careful dosing is crucial to minimize risks. Additionally, their combined antioxidant and anti-inflammatory properties might offer synergistic benefits for healthy aging. However, translating these findings to humans necessitates extensive research. Cannabis comprises diverse compounds with potential therapeutic effects, but direct evidence linking their use to human longevity remains limited. The reviewed studies highlight both the potential benefits and risks of long-term cannabis use in older adults, underscoring the need for further investigation (Table 2).

On one side of the spectrum, studies by Thayer et al. (2019) and Auer et al. (2016) raise concerns about the potential adverse effects of regular cannabis use on cognitive function in aging. Thayer et al. studied adults aged 60 + who had used cannabis weekly for at least the past year (*n* = 28), with an average of 23.55 years of use (SD = 19.89, range 1.5–50 years), comparing them to non-users (*n* = 28). Long-term users showed poorer executive function, essential for planning and decision-making and reduced grey matter, suggesting negative effects on brain health with prolonged use. Similarly, Auer et al. followed a cohort of black and white men and women (aged 18–30 at baseline) over 25 years to assess cumulative cannabis exposure. They found that past cannabis use was linked to poorer verbal memory in middle-aged adults, although other cognitive functions were unaffected (Auer et al. 2016; Thayer et al. 2019).

Furthermore, Burggren et al. found reduced grey matter in hippocampal memory regions among older adults (aged 57–75) who had significant cannabis exposure during adolescence (defined as use on at least 20 days/month before age 20, continuing for at least a year, and minimal use after age 35).(Burggren et al. 2018). These findings emphasize that lifetime cannabis use, particularly when initiated early, may lead to more pronounced cognitive impairments in later life. In contrast, cannabis use that begins or continues into aging might have different or less severe effects. These distinctions underscore the importance of further research to disentangle the cognitive impacts of lifetime use from those of use specifically in aging. However, further research is imperative to comprehensively understand this intricate relationship, accounting for factors such as dosage, duration of use, and individual differences.

Conversely, Watson et al. (2022) revealed increased functional connectivity among the hippocampus, parahippocampal cortex, and anterior lobes of the cerebellum in older adults (aged 60–88, mean age 67.5 years, SD 5.65, 60.6% female) who reported regular cannabis use compared to non-users. The study recruited participants who used cannabis weekly for at least the past year (“users,” *n* = 43) and compared them to “non-users” (*n* = 153). Enhanced connectivity in these regions suggests improved communication and information processing, potentially mitigating age-related declines often associated with weakened functional connectivity. This finding aligns with results observed in animal models, where an increased number of synapses in the hippocampus facilitated memory and learning functions.

However, the heterogeneity within the older cannabis user population, as emphasized by Arora et al., complicates our understanding of the effects of cannabis on aging. He identified three groups of older cannabis users: new users (starting after 60), stop-out/intermittent users, and consistent users. Understanding these groups allows tailored approaches to assess cannabis effects and address potential biases in research, ensuring accurate conclusions. This recognition is vital for developing tailored strategies to assess individual needs and potential health impacts based on unique usage patterns and vulnerabilities (Arora et al. 2021).Additionally, a pilot study by Stypulkowski and Thayer (2022) compared cognitive function between regular cannabis users and nonusers aged 60 and above. The study included a small sample size of 28 regular cannabis users (defined as using at least once per week for the past year) and 10 non-users (defined as those with no more than one lifetime use). Recruitment was conducted via online advertisements and direct mail flyers. While long-term users showed lower executive function compared to non-users and even short-term users, short-term use itself did not appear to have a significant impact on cognitive performance. This suggests a possible window of relative harmlessness for short-term recreational use in this age group. However, the negative association between recent use frequency and working memory performance requires further investigation (Stypulkowski and Thayer 2022).

Lastly, Meier et al. (2022) conducted a longitudinal study on long-term cannabis users (*n* = 86), defined as individuals who used cannabis weekly or more frequently in the past year at age 45 and during earlier assessment waves. These users showed accelerated biological aging and poorer midlife health, financial, and social preparedness for late life compared to non-users. Importantly, these associations were not attributable to childhood socioeconomic deprivation, low IQ, or family history of substance dependence (Meier et al. 2022).This emphasizes the need for further research to inform public health policies and interventions for healthy aging (Meier et al. 2022).

Together, these findings highlight the nuanced effects of cannabis use across different stages of life. Lifetime cannabis use, especially when initiated early, may exacerbate cognitive decline and aging-related vulnerabilities, while cannabis use initiated or continued during aging might produce distinct or less severe outcomes. Further research is essential to disentangle these dynamics and guide informed decision-making regarding cannabis use in older populations.

### Real-world evidence of cannabis-based medicinal products (CBMPs) in aging populations

Despite notable increases in medical cannabis use among older individuals, understanding the characteristics of older patients remains limited due to the exclusion of those over 65 from randomized controlled trials (RCTs) and a lack of high-quality evidence. Real World Evidence (RWE) consistently suggests that CBMPs hold promise in treating various conditions associated with aging, such as insomnia, depression, anxiety, and chronic pain (Lynskey et al. 2023). These are all conditions that may be associated with the aging process, and certainly when co-morbid, may reduce individuals’ quality of life. Furthermore, RWE highlights that CBMPs may also contribute to a reduction in other medications, including opioids, which might also be beneficial for an aging population often prescribed a broad range of medications (Sunderland et al. 2023).

Recent studies have delved into medical cannabis use patterns among older individuals. For instance, Tumati et al. (2022) reported on the characteristics, effectiveness, and side effects of medicinal cannabis in a large sample of older users in Canada, most of whom used it to alleviate pain. They noted improvements in pain, sleep, and mood, with about a third reducing their opioid dosage (Tumati et al. 2022)(Tumati et al. 2022). Data from T21, one of the largest observational studies globally (https://www.drugscience.org.uk/t21/), supports the need to examine older patients using CBMPs, revealing key differences in characteristics and outcomes compared to younger users. Older individuals seeking CBMPs are more likely to be female and cite pain as their primary indication, setting them apart from their younger counterparts.

Crucially, preliminary findings suggest significant health and well-being improvements for older adults prescribed CBMPs, emphasizing the potential of medical cannabis in addressing the unique challenges of aging (Arkell et al. 2023). Additionally, while much of the current focus has been on THC and CBD, other phytocannabinoids, terpenes, and related compounds in cannabis also warrant attention. These secondary compounds, such as cannabigerol (CBG), cannabinol (CBN), and various terpenes, may have unique or synergistic effects that influence outcomes. For example, terpenes like myrcene and limonene have been associated with analgesic, anxiolytic, and anti-inflammatory properties, potentially enhancing the efficacy of cannabinoids (Del Prado-Audelo et al. 2021). Future discussions of CBMPs should consider the broader spectrum of cannabis constituents to fully understand their therapeutic potential and optimize formulations tailored to the needs of aging populations.

### Limitations

While the current evidence in invertebrate and rodent models is encouraging, further research in more complex models and well-designed human trials is crucial to fully understand the potential of cannabinoids including CBD and THC for promoting healthy aging and longevity. Translating findings directly to humans is difficult due to biological differences. Current studies on preclinical trials often use specific doses and delivery methods, and the effects may vary depending on the approach.

Despite intriguing findings in experimental models, several limitations remain. Most studies rely on non-human models, making it difficult to directly translate these findings to humans. The long-term effects of cannabis use, especially negative outcomes, are not well understood. Additionally, the biphasic nature of THC, where low doses may provide benefits such as improved survival and reduced inflammation, while high doses could lead to adverse effects needs further exploration. Other cannabinoids such as CBG CNG and Terpenes, which are abundant in cannabis, play a key role in modulating the overall effects and could potentially modify the impact of THC and CBD on aging-related conditions. Research on how these compounds work together in synergy is crucial for advancing the field.

The human studies also did not control for the route of administration of cannabis, which affects THC blood concentrations (Newmeyer et al. 2016). Future research should account for the route of administration given the high rates of edible use among older adults. Preclinical studies suggest that cannabinoids may have age-dependent effects on brain function and cognition (Thayer et al. 2019). While studies in older animals show potential benefits for cellular repair and reducing inflammation, younger animals may experience contrasting effects. Limited research exists on the impact of cannabis on the aging human brain.

## Conclusion

While the initial excitement surrounding cannabis and its potential for anti-aging is warranted, significant research gaps remain, particularly in differentiating the impacts of lifetime cannabis use from those initiated during aging. Current evidence from human studies highlights that long-term cannabis use, particularly when initiated early in life, may have adverse effects on cognitive function and brain health in older adults. In contrast, limited research suggests that cannabis use initiated in aging might have different outcomes, including potential therapeutic benefits, though this remains to be conclusively determined.

Promising findings from experimental models underscore the importance of further exploration to clarify the mechanisms underlying cannabinoids’ effects on aging, neuroprotection, and inflammation. These models reveal the biphasic effects of THC, where low doses may promote survival and reduce inflammation, while higher doses could lead to negative outcomes. Such insights can inform the development of more precise and effective cannabis-based therapies for age-related conditions.

Real-world evidence also emphasizes the growing utilization of medical cannabis by older adults, driven by age-related health changes and evolving policies. To address this trend responsibly, there is an urgent need for rigorous clinical trials and longitudinal studies that consider diverse use patterns, dosages, and interactions with common health conditions and medications in aging populations.These findings open exciting avenues for exploring novel therapeutic interventions for age-related cognitive decline and neurological disorders.

Research must also delve into the roles of other cannabinoids, such as CBG (cannabigerol) and CBN (cannabinol), along with terpenes, both in isolation and in combination. Exploring the synergy between cannabinoids and other compounds, like omega-3 fatty acids, could offer innovative strategies for promoting brain health and longevity.

It’s also important to emphasize that healthy aging is a complex process influenced by various factors, and cannabinoids like CBD and THC alone are unlikely to serve as a singular solution. While these compounds show promise in addressing specific age-related challenges, maintaining a healthy lifestyle through a balanced diet, regular exercise, good sleep hygiene, and stress management remains crucial for promoting healthy aging and overall well-being.

## Data Availability

Not applicable.

## Declarations

### Authors information

NS is a Consultant Psychiatrist and a medicinal cannabis prescriber in Lenus Global and his private practice. SN is a Senior DNA Scientist at Source Bioscience Cambridge, UK. AKS is a scientific advisor to Somai Pharmaceuticals and Evolve. MB serves as a Director at Maple Tree and is a Consultant Neurologist in the company.

## Abbreviations

ECS: Endocannabinoid system
CBD: Cannabidiol (CBD)
THC: Δ^9^-tetrahydrocannabinol
CBG: Cannabigerol
CBN: Cannabinol
mTOR: Mechanistic Target of Rapamycin
CBMPs: Cannabis-Based Medicinal Products
FAAH: Fatty acid amide hydrolase
PPAR: Peroxisome Proliferator-Activated Receptors
CB1R: Cannabinoid Receptor 1
TRNs: Touch Receptor Neurons
RNAi: RNA interference
SIRT1: Sirtuin 1 (a longevity-related protein)
DOHaD: Developmental Origins of Health and Disease
F1 generation: First filial generation (offspring of the original experimental animals)
WE: Real World Evidence
RCTs: Randomized Controlled Trials
